# Individual Differences in Slow-Wave-Sleep Predict Acquisition of Full Cognitive Maps

**DOI:** 10.3389/fnhum.2018.00404

**Published:** 2018-10-08

**Authors:** Itamar Lerner, Mark A. Gluck

**Affiliations:** Center for Molecular and Behavioral Neuroscience, Rutgers University-Newark, Newark, NJ, United States

**Keywords:** sleep, slow-wave-sleep, SWS, memory consolidation, insight, virtual navigation, memory replay

## Abstract

Accumulating evidence suggests that sleep, and particularly Slow-Wave-Sleep (SWS), helps the implicit and explicit extraction of regularities within memories that were encoded in a previous wake period. Sleep following training on virtual navigation was also shown to improve performance in subsequent navigation tests. Some studies propose that this sleep-effect on navigation is based on explicit recognition of landmarks; however, it is possible that SWS-dependent extraction of implicit spatiotemporal regularities contributes as well. To examine this possibility, we administered a novel virtual navigation task in which participants were required to walk through a winding corridor and then choose one of five marked doors to exit. Unknown to participants, the markings on the correct door reflected the corridor’s shape (from a bird’s eye view). Detecting this regularity negates the need to find the exit by trial and error. Participants performed the task twice a day for a week, while their overnight sleep was monitored. We found that the more time participants spent in SWS across the week, the better they were able to implicitly extract the hidden regularity. In contrast, the few participants that explicitly realized the regularity did not rely on SWS to do so. Moreover, the SWS effect was strictly at the trait-level: Baseline levels of SWS prior to the experimental week could predict success just as well, but day-to-day variations in SWS did not predict day-to-day improvements. We propose that our findings indicate SWS facilitates implicit integration of new information into cognitive maps, possibly through compressed memory replay.

## Introduction

Considerable evidence from the past two decades suggests that sleep, particularly Slow-Wave-Sleep (SWS), facilitates a variety of hippocampal-dependent cognitive processes (Rasch and Born, 2013). One example of such effect is the development of cognitive maps in humans. Studies show that sleep following practice on a virtual navigation task improves performance on the same task the next waking period (e.g., Peigneux et al., 2004; Wamsley et al., 2010; Nguyen et al., 2013) and that activity patterns in the hippocampus during practice are repeated during SWS and predict later improvement (Peigneux et al., 2004). Moreover, artificially eliciting brain-activity patterns from the practice period during sleep stages N2 and SWS using targeted memory reactivation (TMR) techniques improve next-morning performance even further (Shimizu et al., 2018).

The mechanisms by which SWS contributes to navigation performance are not entirely clear. Previous experiments have often included conspicuous landmarks along the navigated route and, indeed, there is evidence to suggest that the sleep effect depends on explicit recognition of those landmarks rather than on implicit development of a full cognitive map (Noack et al., 2017). This possibility corresponds with contemporary understanding of SWS as a state in which previously encoded memories are reactivated in the hippocampus (“memory replay”) to support memory consolidation (Diekelmann and Born, 2010). In the case of navigation tasks, such replay may help to consolidate landmark memories within their spatial context and thus yield sleep-dependent facilitation in performance. Nevertheless, it is well recognized that navigation behavior depends on various cognitive abilities, some of which are implicit (Wolbers and Hegarty, 2010), and therefore the actual effect of sleep may be multifaceted. There is considerable evidence from other paradigms that memory replay during SWS does not only support the simple strengthening of memories, but also helps extracting regularities embedded in those memories, including implicit and explicit detection of sequential patterns (Fischer et al., 2006; Durrant et al., 2011; Wilhelm et al., 2013), identifying linguistic regularities in words and sentences (Gomez et al., 2006; Batterink and Paller, 2017), and gaining insight into temporal contingencies (Wagner et al., 2004; Yordanova et al., 2012). Such sleep-dependent extraction of regularities could potentially underlie improvement in navigation performance as well, possibly because it can facilitate the integration of new information pertaining to a spatial environment into a cognitive map.

Here, we hypothesized that sleep may facilitate implicit and explicit development of a full cognitive map during navigation in a virtual 3D environment that does not contain any conspicuous landmarks en route (and thus does not rely on simple memory recall). To that end, we developed a novel virtual navigation task in which performance improvement crucially depends on (implicit or explicit) understanding of the whole traveled route rather than memorization of any specific highlighted spots. In addition, following our previous study showing that effects of sleep on learning might be more trait- than state-dependent (Lerner et al., 2016), we employed a multiple-night paradigm that differentiated effects to those depending on daily sleep patterns and those depending on average sleep patterns characterizing the individual as a whole.

## Methods

### Participants and Design

**FIGURE 1:**
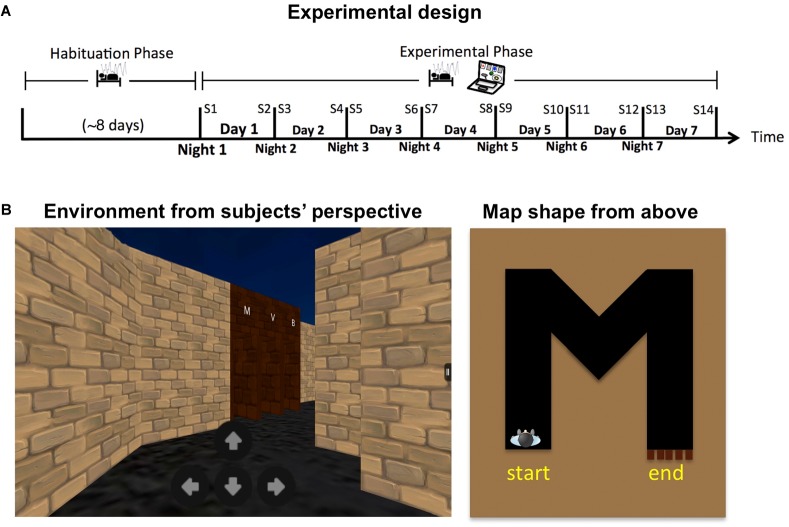
**(A)** Experimental design. **(B)** Example of the navigational task. Left: View from the subject’s perspective. Right: Top (“bird’s eye”) view of a full route during a single trial.

Twenty healthy students (mean age: 22.15; *SD* = 2.5; four females) participated in the study for monetary compensation (**Supplementary Table S1**). During the study they maintained their regular caffeine intake and refrained from alcohol consumption and daytime napping. Long-term sleep monitoring and administration of the behavioral task followed an established protocol previously used by us in a former study (see Lerner et al., 2016 and **Supplementary Material**). In short, participants first monitored their sleep at home using a wireless sleep-monitoring headband (Zeo Inc., Newton, MA, United States) and an actigraphy bracelet (Micro Motionlogger Sleep Watch, AMI, Ardsley, NY, United States) for a habituation period of at least 5 days (mean: 7.75; *SD* = 2.5). They then continued to monitor their sleep for an additional week, during which they also practiced on a virtual navigation task twice a day, in the morning (half an hour after waking up) and in the evening (within an hour before going to sleep) using a Kindle tablet (Amazon.com Inc., Seattle, WA, United States). Behavioral data from the task was sent automatically to a secured email after each session, for a total of 14 sessions (see **Figure 1A** for task design).

In each trial of the virtual navigation task, participants were placed at the beginning of a winding corridor. They needed to navigate their way to the end of the corridor using four on-screen buttons (forward, backward, turn right, turn left; **Figure 1B**). There, they faced five doors, each marked above with a different letter or a digit. One door led to the exit, while the others were locked. Participants needed to find the correct door by trial and error, and were instructed to try to exit each corridor as quickly as possible. Once outside, the next trial began with a new corridor. Unknown to participants, the markings above the correct door always reflected the shape of the corridor they have just traversed (from a bird’s eye view). Realizing this consistency, either implicitly or explicitly, allowed participants to avoid trying all the doors one by one and thus reduce the number of failed attempts.

There were four trials in each session, for a total of 56 trials along the week. Twenty-eight different letters/digits were used as targets and distractors, with each being the correct answer exactly twice across the 56 trials. The order of targets, the location of the correct door and the identity of distractors were randomized across trials uniquely for each participant, under the constraint that letters/digits with very different shapes were used in each trial to avoid confusion (see **Supplementary Material** for examples). Following the last session, participants filled in a questionnaire, asking them if they had realized the significance of the markings above the doors, and if so – at what point along the experiment had they realized it.

### Data Analysis

We measured behavioral performance as the number of different incorrect doors participants attempted to open in each trial before finding the correct one (ranging from 0 to 4). We computed participant’s “Average Error” for each session as the average number of incorrect attempts over the four trials for that session, yielding 14 values for each participant.

Statistical analysis followed our previous long-term sleep study (Lerner et al., 2016; please see the **Supplementary Material** for more details). Data from the two sleep monitoring devices were integrated for each subject to yield a measure of total time spent in each sleep stage of each experimental night, as well as during the habituation phase. These devices were able to distinguish between three sleep stages: SWS (equivalent to N3 sleep stage in the literature), rapid eye movement (REM) sleep, and a “Light sleep” stage equivalent to the aggregation of N1 and N2 sleep stages. These measures were then used in a mixed-model Analysis of Variance (ANVOA) to predict the number of errors. The basic ANOVA model included the average time spent in each sleep stage for each subject as “between-subject” factor to assess the influence of individual trait-level differences, as well as the daily deviations of each sleep stage from the individual average as a “within-subject” factor, to assess the contribution of state-level fluctuations in sleep patterns from one night to the other (with each daily sleep deviation value corresponding to performance the following day). Additional within-subject factors were Day (1–7) and Time of day (Morning and Evening), and their interaction. Covariance structure for errors was defined by Kronecker products with unstructured covariances for Time and first-order autoregressive AR(1) for Day. Analysis was performed using SAS Studio 3.71 (SAS Institute). Similar follow-up analyses are described in Results.

A second analysis focused on examining the relation between trait-level sleep measures and global variables characterizing learning in the task as a whole. To that end, we fitted a learning curve for each participant’s scores, using a decaying exponential function described by *F*(*t*) = *Ae*^−*bt*^, with *t* = 1...14, and *A*, *b* being positive parameters fitted for each individual. Global learning performance was characterized for each participant as the final (i.e., *t* = 14) value on that curve^1^. We then ran a multiple regression model with the individual average time in each sleep stage as three predictors of the global learning performance measure. Analysis was performed using Matlab 2017a (MathWorks).

## Results

Across subjects, behavioral and sleep data were collected successfully in 95% and 88% of all sessions and nights, respectively. Missing values of the learning data were filled in by linear extrapolation based on the nearest neighboring sessions for each subject, whereas unrecorded sleep data were treated as missing values.

**FIGURE 2:**
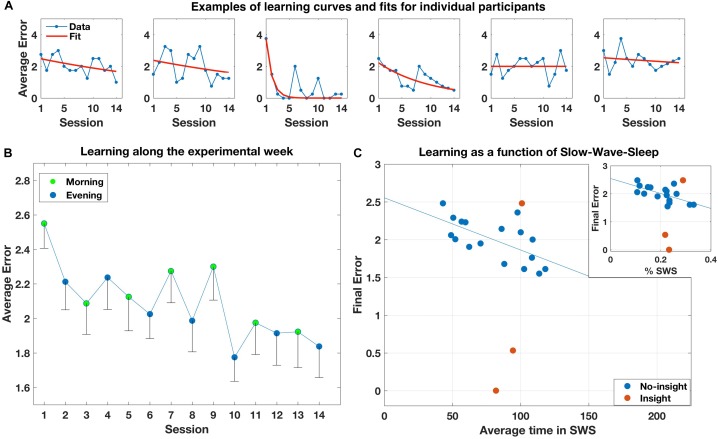
**(A)** Examples of individual learning curves and exponential fits of six participants, representing different learning abilities (moderate learning without insight into the maps structure; learning with insight; little or no learning). **(B)** Average error rates across participants along the experimental sessions. Error bars represent standard deviation of the mean. **(C)** Final error rates at the end of learning for all participants as a function of their individual average time in SWS. Blue dots represent participants that did not gain insight into the maps’ structure. Brown dots represent participants that gained insight. Inset: Same, for average individual proportion of SWS out of total sleep time.

Average performance for the 14 sessions is shown in **Figure 2B**. A one-way repeated-measure ANOVA with Session as a single factor showed a significant improvement [*F*(13,247) = 1.948, *p* = 0.026]. Individual performance on the task was, however, quite variable both between- and within-subjects (see **Figure 2A** for typical examples). Only three participants reported having an insight into the hidden regularity. Two had the insight on the first and third day, respectively, and showed markedly improved performance on the task that corresponded with their estimated time of insight. The third participant reported having insight only the fifth day, and did not show a corresponding behavioral improvement. All other participants did not explicitly discover the regularity, but most still showed moderate implicit improvement on the task along the week.

Results of the mixed model ANOVA did not reveal any within- or between-subject effects of sleep on performance, with only Day showing a marginal effect [*F*(6,97) = 1.83, *p* = 0.102; **Supplementary Table S2**]. We subsequently ran a similar ANOVA but this time examining the effects of sleep on the difference between performance in the morning and performance in the previous evening (with Day as a single within-subject factor, in addition to the same within and between-subject sleep factors. There were only six levels of Day given that the dependent measure was difference scores). We found a significant effect of baseline N1/N2 sleep, such that more individual N1/N2 was associated with higher increase in errors from the evening to the following morning [*F*(1,16) = 5.27, *p* = 0.036; **Supplementary Table S3**]. No other effects were significant. To examine whether this effect is maintained when controlling for total sleep time, we followed up this analysis by running a second, similar ANOVA with % N1/N2 (proportion of time in N1/N2 out of total sleep time) as a single factor (both within and between subjects as before). However, no effects were significant (**Supplementary Table S4**). Finally, we ran a third ANOVA for the difference scores with total sleep time (within- and between-subjects) as a single factor. Again, no effects were significant (**Supplementary Table S5**).

Moving on to global measures of performance, multiple regression analysis of the effect of average time in each sleep stage on the final error rates was not significant. However, visual inspection revealed that the three participants that had insight–particularly the two that showed marked performance improvement–were obscuring a clear effect of SWS on performance for the other participants. We therefore reran the multiple regression model with only the 17 non-insight participants. This time, the model effect was significant [*F*(3,13) = 4.29, *p* = 0.026], driven by a negative correlation with SWS (*p* = 0.013; **Figure 2C**): The more average SWS subjects had, the less errors they performed. To follow up this analysis, we computed the Pearson correlation between the percent of time in SWS with the final error. Again, the effect was highly significant (*r* = −0.65, *p* = 0.0047; **Figure 2C**, inset). Re-running the analysis with total sleep time as a single predictor was not significant (*p* = 0.931).

Next, to verify that the effects did not depend upon the exponential fitting, we computed the Pearson correlation between SWS and % SWS and the average error of each subject over the last 2 days instead of the final error value predicted by the exponential fit, and found even stronger effects (*r* = −0.748, *p* < 0.001 and *r* = 0.698, *p* = 0.002, for SWS and % SWS, respectively). Next, we reran the analysis using the average baseline levels of SWS from the habituation period, instead of the average amount over the experimental period. Again, effects remained strongly significant for both SWS and % SWS (*r* = −0.684, *p* = 0.002, and *r* = −0.639, *p* = 0.006, respectively). Finally, we reran the analysis with all 20 participants, but instead of using the final error for the three insight participants, we used the values that fit their performance curves just before they had insight (middle of days 1,3, and 5, respectively, for each participant). Again, the SWS effect remained significant (*r* = −0.488, *p* = 0.029) while % SWS was marginally significant (*r* = −0.392, *p* = 0.087), suggesting that the very gain of insight might dramatically change performance that was affected by SWS until that time.

## Discussion

Our main finding in the current study is that the more time individuals spend in SWS on average, the better they are able to develop implicit cognitive maps of recently traversed environments. Specifically, unlike previous works, this effect was found in the absence of any conspicuous landmarks along the traveled routes, suggesting it did not rely on sleep improving simple recall of contextual cues (cf. Noack et al., 2017). In contrast, gaining explicit insight into the structure of these maps seems to depend on additional mechanisms beyond SWS. This dichotomy between explicit and implicit effects of SWS in regards to insightful behavior echoes previous findings in the literature, in which SWS was shown to encourage implicit detection of sequential regularities while explicit detection of these regularities required additional processes (Yordanova et al., 2010).

One mechanism that could potentially account for our results is sleep-dependent “temporal scaffolding,” previously suggested by us to explain the general effect of sleep on extraction of hidden regularities within newly learned stimuli (Lerner, 2017a,b). Noting that the effects of sleep are often achieved when the regularities to be extracted are temporal (i.e., when an event at one time point consistently predicts another event occurring a few seconds later), we hypothesized that one specific property of memory replay during SWS could underlie this effect: its time-compressed nature. Specifically, memory replay during SWS is known to occur in an accelerated form compared to the original experience (Rasch and Born, 2013). This acceleration may allow bridging temporal gaps between events and create representations of the sequential experiences that are stripped of their temporal component. Consequently, Hebbian mechanisms, which typically have a timescale of only 50–100 ms (August and Levy, 1999), could pick up temporal regularities embedded in those representations that were previously too distant in time to be detected. The theory suggests that cortical mechanisms could then exploit those new hippocampal representations achieved during sleep to allow gaining explicit insight into the regularities. Applying the model to the current results, we suggest that compressed replay of a few-seconds worth of travel in the virtual environment could have helped form time-free representations of those (partial) routes. Consequently, these representations were implicitly elicited during task performance the next waking period, biasing participants to choose the doors with the markings corresponding to those representations. The more SWS participants tend to have on average, the more opportunities there will be for compressed replay of those routes. Consistent with this view, previous human and rodent studies suggested that memory replay (albeit during quiet wake rather than sleep) plays an active role in flexible learning of cognitive maps, and not just in their consolidation (e.g., Gupta et al., 2010; Craig et al., 2016). Nevertheless, inconsistent with our theory, explicit detection of the regularity in our task seems to have depended on additional, non-SWS mechanisms, though strong conclusions cannot be drawn due to the small number of participants gaining insight.

We also found evidence of an N1/N2 effect on average performance. This result resembled our previous long-term sleep study using the same methodology but a different learning paradigm (Lerner et al., 2016). Others have also implicated N2 in the facilitation of navigational performance (Wamsley et al., 2010; Shimizu et al., 2018). N2 shares several physiological characteristics with SWS (e.g., sleep spindles) and both stages are sometimes grouped together as non-REM sleep; however, it is currently not clear if the mechanisms contributing to the N2 and SWS effects on memory are similar. Since in the current study the N1/N2 effect was evident only for raw average times in N1/N2 but not when using a relative measure (% N1/N2 out of total sleep time), we did not pursue it further.

Finally, why didn’t day-to-day sleep affect day-to-day changes in performance? One possibility is that within-subject performance was too noisy to allow detecting small sleep effects. However, other results from our lab suggest otherwise. Particularly, in a small follow-up experiment (**Supplementary Material**), participants performed all of the 56 trials in two sessions with a 90-min nap (or quiet rest) in between, and hardly any participant showed any improvement. Therefore, it seems that a single sleep session is not sufficient to induce facilitation in this task. This replicates our previous results using a different learning task (Lerner et al., 2016) and suggests that sleep, and SWS in particular, might have an accumulated effect over multiple nights. Together, those earlier and current results point to the possibility that performance in any task that benefits from replay could be positively correlated with individual levels of SWS, especially if the task is not straightforward and requires long periods of learning.

Our results, however, need to be interpreted with some caution. First, the implicit measure of performance, error rates, is an indirect measure of spatial knowledge. In principle, error rates could have improved by developing abilities that are not strictly based on navigation (e.g., counting the number of turns on the route and choosing a symbol with a number of edges that match it, and so on). Incorporating a second measure of spatial knowledge, such as pointing toward the starting point at the end of each trial (e.g., Craig et al., 2016), may help verify our conclusions in future studies. Second, as with any correlational finding, one could not naively infer a causal relationship. Specifically, as discussed in Lerner et al. (2016), the relation between SWS and task performance could also be indirect, stemming from a correlation of each of them with a third variable, such as attention or mindset flexibility. Moreover, time spent in SWS is a crude measure of the mechanisms involved in memory consolidation, and does not directly tap memory replay. Further research, manipulating SWS over multiple nights and measuring brain activation during performance, would be needed to establish a direct causal relation of SWS and performance in our task and provide further support for the potential involvement of memory replay in the process.

## Data Availability

The raw data supporting the conclusions of this manuscript will be made available by the corresponding author, without undue reservation, to any qualified researcher upon request.

## Ethics Statement

This study was carried out in accordance with the recommendations of the Rutgers University Institutional Review Board with written informed consent from all subjects. All subjects gave written informed consent in accordance with the Declaration of Helsinki. The protocol was approved by the Rutgers University Institutional Review Board.

## Author Contributions

IL conceived the task, designed the experiments, analyzed the results, and wrote the paper. MG oversaw the study and contributed to the paper.

## Conflict of Interest Statement

The authors declare that the research was conducted in the absence of any commercial or financial relationships that could be construed as a potential conflict of interest.

## Footnotes

1 In Lerner et al. (2016) we also used a second global parameter, the time it took participants to reach learning plateau. This was not attempted here because very few participants reached a plateau.
