# Supplementary material for: Individual Differences in Slow-Wave-Sleep Predict Acquisition of Full Cognitive Maps
Source: Front Hum Neurosci. 2018 Oct 8;12:404. doi: 10.3389/fnhum.2018.00404 (PMC6186812; doi:10.3389/fnhum.2018.00404)
Supplement: Supplementary file 1 [file Data_Sheet_1.docx]

Supplementary Material

**Individual Differences in Slow-Wave-Sleep Predict Acquisition of Full Cognitive Maps**

Itamar Lerner*, Mar A. Gluck

*** Correspondence:** Corresponding Author: itamar.lerner@gmail.com

**1. Supplementary Materials for Methods**

**1.1 Table S1.** *Summary of Demographics and Sleep Parameters*

|  | *Mean (SD)* |
| --- | --- |
| **Demographics** |  |
| Age (years) | 22.15 (2.5) |
| Education (years completed) | 15.65 (1.8) |
| **Sleep Parameters** |  |
| Habituation |  |
| Number of Nights | 7.75 (2.5) |
| Total Sleep Time (TST, minutes) | 419.9 (59.1) |
| Time in N1/N2 (minutes) | 199.5 (39.4) |
| Time in SWS (minutes) | 85.8 (22.5) |
| Time in REM (minutes) | 134.6 (26.5) |
| Ratio N1/N2 out of TST | 0.472 (0.05) |
| Ratio SWS out of TST | 0.210 (0.06) |
| Ratio REM out of TST | 0.319 (0.05) |
| Experimental |  |
| TST (minutes) | 397.7 (48.9) |
| Time in N1/N2 (minutes) | 187.5 (34.6) |
| Time in SWS (minutes) | 82.2 (24.5) |
| Time in REM (minutes) | 127.9 (25.5) |
| Ratio N1/N2 out of TST | 0.470 (0.05) |
| Ratio SWS out of TST | 0.211 (0.07) |
| Ratio REM out of TST | 0.318 (0.05) |

**1.2 Additional Details Regarding Data Collection and Statistical Analysis**

**1.2.1 Sleep Monitoring System**

Sleep data was collected using a mobile sleep monitoring system that included an automated wireless sleep-monitoring headband (Zeo Inc., Newton, MA), an actigraphy bracelet (Micro-MotionLogger Sleep watch, Ambulatory Monitoring, Inc., Ardsley, NY), and an Android tablet (Amazon.com, Inc., Seattle, WA). In addition, participants were instructed to keep a sleep log noting their sleep/wake times and any nocturnal awakenings throughout the experiment. The sleep-monitoring headband is equipped with a single bi-polar fabric sensor that transmits data wirelessly to the Android tablet, which acts as a base station. The sensor is fitted with three silver-coated electrodes used to detect brain waves (EEG), eye movements (EOG), and the movement of the frontalis muscle (EMG). The signals from these electrodes are analyzed in real time to produce sleep staging in 30-second epochs. This sleep staging, the accuracy of which was validated for nocturnal sleep compared to PSG in multiple studies (e.g., Griessenberger et al., 2013; Shambroom et al., 2012), is a reduced version of the official staging criteria by the American Association of Sleep Medicine (Iber, et al., 2007) and differentiates between four stages rather than five — wake, N1/N2 (combined N1 and N2 stages, termed ‘Light sleep’), Slow Wave Sleep (SWS; also called ‘Deep sleep’), and Rapid-Eye-Movement (REM) sleep. The actigraphy bracelet is a research-grade device that contains a built-in accelerometer used to infer sleep/wake decisions in one-minute epochs based on participants’ arm movements (Ancoli-Israel et al., 2003; de Souza et al., 2003). Participants wore the actigraph on the non-dominant wrist throughout the entire study. Data was extracted from the devices at the end of the experiment, and was used to assess the sleep/wake validity of the sleep-monitoring headband. Preparation of the sleep data for statistical analysis was conducted in Matlab R2015a (Mathworks Inc.). Epoch-by-epoch sleep data for both the sleep-monitoring headband and the actigraphy bracelet were extracted from the devices while maintaining the original time stamps for each. The actigraphy data was analyzed using the Action4 software program (AMI, Ardsley, NY), which inferred sleep/wake decisions for the entire period based on the Cole-Kripke algorithm (Cole et al.,1992). After time-aligning the data from both of the devices, the sleep/wake agreement between them was compared based on visual inspection (treating all specific sleep stages from the sleep-monitoring headband as “sleep”; see Lerner et al., 2016 for demonstrations). Nights with low agreement between the devices (typically because of missing segments of data for a given night due to the headband moving from its optimal position on the forehead, or from significant discrepancies between the initiation/termination of sleep on a given night between the two devices) and/or between the devices and the participants’ sleep logs, were removed.

**1.2.2 Statistical Analysis Using the Mixed- Model ANOVA**

Data from the sleep-monitoring headband and the actigraphy bracelet were integrated for each subject as described above to yield measures of total time spent in each sleep stage at each night. These measures were then used in a mixed-model Analysis of Variance (ANOVA) to predict the daily behavioral measure in the task. The dependent variable for the model was the average error (as described in the main text) for each session, for each participant, across all 14 sessions (7 days x 2 sessions/day). In order to assess if the average error was affected by stable, individual differences in sleep parameters, the ANOVA model included the average time spent in REM, SWS and N1/N2 (across all experimental nights) as “between-subjects” factors. To assess whether “within-subject” fluctuations in sleep patterns from one night to another affected the dependent variables, the daily deviations of REM, SWS and N1/N2 of each participant from his/her corresponding averages were entered as continuous (centered) covariates crossed with blocks (i.e., participants). The sleep variables of each night (termed REMcovdev, SWScovdev and N1/N2covdev in Table S2) were compared with the Average Error of the following day. Additional within-subject factors included Day (1 to 7) and Time of day (morning vs. evening). Finally, the Day x Time interaction was also included. Comparisons of within- and between-subjects effects were carried out in SAS 9.3 (SAS Institute) using the *Mixed* procedure with a covariance structure for error defined by Kronecker products, specifying unstructured covariances for the Time-of-day (mornings vs. evening) factor and a first-order autoregressive AR(1) structure for Day. A follow up analysis, examining the difference between daily morning performance and the evening preceding it, was carried out similarly but omitting Time of day as a factor (Table S3). Finally, two similar follow-up analyses were carried out with a single sleep variable, proportion of time spent in N1/N2 out of total sleep time (Table S4), and Total sleep time (Table S5).

**1.2.3 Symbols Used for Map Shapes and Distractors**

The 28 digit/letter symbols used in the navigation task were: 0, 1, 2, 3, 4, 5, 6, 7, 8, a, c, e, g, G, h, J, L, m, M, n, O, q, r, S, U, V, W, Z. In each trial, the marked doors at the end of a corridor always included, in addition to the correct answer, symbols that are different enough from each other to reduce the likelihood of confusion. For example, O never appeared with 0 or 6; M never appeared with m, Z or W; q never appeared with g or 6, U did not appear with V, and so on.

**2. Supplementary Materials for Results**

**Table S2.** *Results of a Mixed Model ANOVAs for the error rates across the experimental week in the virtual navigation task.*

| *Effect* | *Num DF* | *Den DF* | *F Value* | *p* |
| --- | --- | --- | --- | --- |
| REM | 1 | 16 | 0.17 | 0.6893 |
| SWS | 1 | 16 | 2.00 | 0.1768 |
| N1/N2 | 1 | 16 | 0.50 | 0.4906 |
| Time | 1 | 19 | 1.81 | 0.1941 |
| Day | 6 | 97 | 1.83 | 0.1018 |
| Time*Day | 6 | 97 | 1.04 | 0.4070 |
| REMcovdev | 1 | 210 | 1.25 | 0.2639 |
| SWScovdev | 1 | 210 | 0.04 | 0.8381 |
| N1/N2covdev | 1 | 210 | 0.01 | 0.9272 |

*Note.* Mixed-model ANOVA for error rates including tests of fixed effects and continuous-covariate sleep measures. Sleep variables termed “covdev” represent the within-subject daily deviations from the individual subject’s mean in the corresponding sleep stage. *Num DF*: Numerator Degrees of Freedom; *Den DF*: Denominator Degrees of Freedom. Significant/marginally significant values are highlighted in grey.

**Table S3.** *Results of a Mixed Model ANOVA for morning-evening difference scores in error rates across the experimental week in the virtual navigation task.*

| *Effectdddd* | *Num DF* | *Den DF* | *F Value* | *p* |
| --- | --- | --- | --- | --- |
| REM | 1 | 16 | 0.50 | 0.4909 |
| SWS | 1 | 16 | 0.48 | 0.4966 |
| N1/N2 | 1 | 16 | 5.27 | 0.0356 |
| Day | 5 | 76 | 0.86 | 0.5149 |
| REMcovdev | 1 | 76 | 0.81 | 0.3701 |
| SWScovdev | 1 | 76 | 0.09 | 0.7661 |
| N1/N2covdev | 1 | 76 | 0.00 | 0.9469 |

*Note.* Significant values are highlighted in grey.

**Table S4.** *Results of a Mixed Model ANOVA for the Effects of Proportional N1/N2 on morning-evening difference scores in error rates.*

| *Effectdddd* | *Num DF* | *Den DF* | *F Value* | *p* |
| --- | --- | --- | --- | --- |
| pN1/N2 | 1 | 18 | 2.33 | 0.1443 |
| Day | 5 | 78 | 0.78 | 0.5643 |
| pN1/N2covdev | 1 | 78 | 0.39 | 0.5340 |

*Note.* pN1/N2 refer to proportional (%) N1/N2 values.

**Table S5.** *Results of a Mixed Model ANOVs for the Effects of total sleep time (TST) on morning-evening difference scores in error rates.*

| *Effectdddd* | *Num DF* | *Den DF* | *F Value* | *p* |
| --- | --- | --- | --- | --- |
| TST | 1 | 18 | 2.46 | 0.1340 |
| Day | 5 | 78 | 0.90 | 0.4871 |
| TSTcovdev | 1 | 78 | 0.70 | 0.4053 |

*Note.* TST refers to Total Sleep Time.

**3. Supplementary Materials for Discussion**

We tested nine additional participants performing the same navigational task described in the main text, but with the task administered in two sessions with only one break in between. Participants performed 28 trials at the afternoon (~1:00pm), followed by a two-hour intermission during which they were either allowed a 90-minute nap, or stayed awake to watch a movie. Following the intermission (with an extra half an hour break to avoid sleep inertia in the sleep group), participants performed additional 28 task trials, thus completing the full 56 trials as participants in the main experiment. Other than the administration schedule, the task was identical to the one described in the main text. Like in the main experiment, participants later reported whether they gained insight into the hidden regularity. There were a total of 5 sleeping participant and 4 wake participants, with similar demographics to the ones in the main experiment. In contrast to the main experiment, however, the individual and average learning curves showed that hardly any participant improved performance (Figure S1), and no participant reported gaining insight into the cognitive structure of the maps in the follow-up questionnaire.


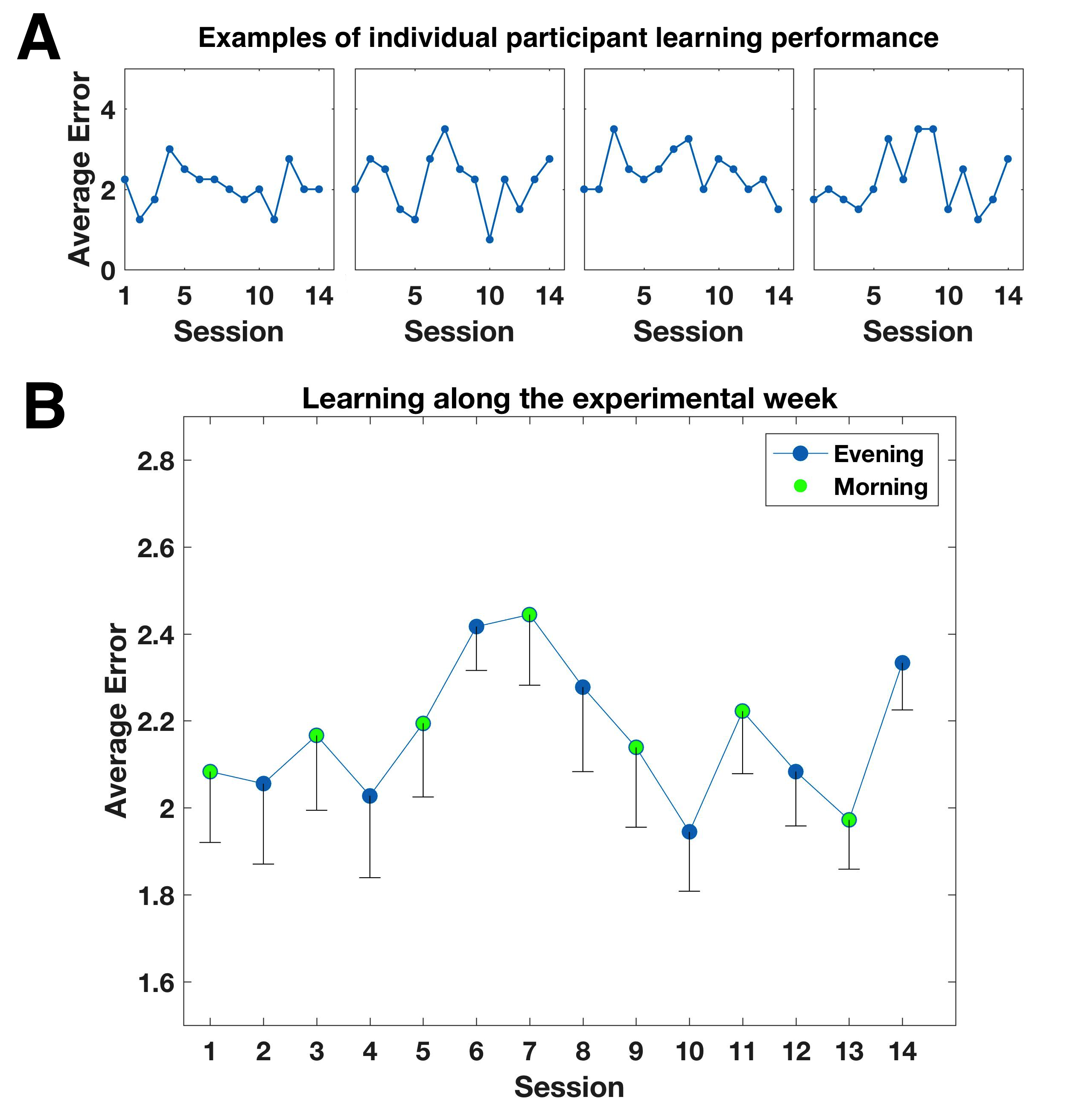


Figure S1. A. Examples of typical learning curves of participants in the nap study. B. Average error rates across participants in the nap study along the experimental sessions. Error bars represent standard deviation of the mean.

**References**

Ancoli-Israel, S., Cole, R., Alessi, C., Chambers, M., Moorcroft, W., & Pollak, C. (2003). The role of actigraphy in the study of sleep and circadian rhythms. American Academy of Sleep Medicine Review Paper. *Sleep, 26*(3), 342-392.

Cole, R. J., Kripke, D. F., Gruen, W., Mullaney, D. J., & Gillin, J. C. (1992). Automatic sleep/wake identification from wrist activity. *Sleep, 15*(5), 461-469.

de Souza, L., Benedito-Silva, A. A., Pires, M. N., Poyares, D., Tufik, S., & Calil, H. M. (2003). Further validation of actigraphy for sleep studies. *Sleep, 26*(1), 81-85.

Griessenberger, H., Heib, D., Kunz, A., Hoedlmoser, K., & Schabus, M. (2013). Assessment of a wireless headband for automatic sleep scoring. *Sleep and Breathing, 17*(2), 747-752. doi: 10.1007/s11325-012-0757-4

Iber, C., Ancoli-Israel, S., Chesson, A., & Quan, S. (2007) The AASM manual for the scoring of sleep and associated events: rules, terminology, and technical specifications, 1^st^ ed. Westcherster, Illinois: Amercian Academy of Sleep Medicine.

Lerner, I., Lupkin, S. M., Corter, J. E., Peters, S. E., Cannella, L. A., & Gluck, M. A. (2016). The influence of sleep on emotional and cognitive processing is primarily trait- (but not state-) dependent. *Neurobiology of Learning and Memory, 134* (2016), 276-286.

Shambroom, J. R., Fabregas, S. E., & Johnstone, J. (2012). Validation of an automated wireless system to monitor sleep in healthy adults. *Journal of sleep research, 21*(2), 221-230. doi: 10.1111/j.1365-2869.2011.00944.x
